# Investigation of Beclin 1 and TNF-α expressions in preeclampsia placentas: Immunohistochemical study

**DOI:** 10.1097/MD.0000000000034757

**Published:** 2023-08-18

**Authors:** Ece Öcal, Veysel Toprak, Senem Alkan Akalin, Firat Aşir, Engin Deveci

**Affiliations:** a Divison of Perinatology, Antalya Research and Education Hospital, Antalya, Turkey; b Department of Gynecology and Obstetrics, Eyyübiye Education and Research Hospital, Şanliurfa, Turkey; c Divison of Gynecology and Obstetrics, Private Medical Practice, Diyarbakir, Turkey; d Department of Histology and Embryology, Faculty of Medicine, Dicle University, Diyarbakir, Turkey.

**Keywords:** Beclin1, normotensive, placenta, preeclampsia, TNF-α

## Abstract

**Background:**

Preeclampsia is a pregnancy complication Aim of this study was to investigate expression of Beclin1 and tumor necrosis factor (TNF)-α in normotensive and preeclamptic placentas of pregnant women patients.

**Methods:**

Twenty normotensive and 20 preeclamptic patients placentas were dissected for paraffin- wax processing. Placental samples were embedded in parafin blocks. Sections were stained with Hematoxylin-Eosin staining and TNF-α and Beclin1 immunostaining.

**Results:**

In control group, root and floating villi were normal in histological perspectives, syncytial node number was low, vessels were normal with connective tissue. No hemorrhage was observed in the intervillous area. In preeclampsia group, decidual cell degeneration and fibrinoid accumulation increased. Vascular dilatation and congestion with mononuclear cell infiltration were observed. Beclin1 reaction was generally negative in control group. In preeclampsia group, Beclin1 reaction was increased in decidual cells, syncytial nodes and bridges and in chorionic villi and in some Hoffbauer cells. In control group, TNF-α expression was mainly negative but only in some decidual cells. In preeclampsia, TNF-α reaction was observed in degenerated decidua cells, in leukocytes and in villi.

**Conclusion:**

In preeclampsia placentas, degenerated decidua cells and inflammation increased. It was thought that Beclin1 and TNF-α signals could be used as a marker in affecting the fetal structure of blood flow in preeclamptic placentas.

## 1. Introduction

Maternal morbidity is a general term that defines any health problem related to pregnancy complications gestational diabetes mellitus and hypertension, preeclampsia, infections and hemorrhage. Regular clinical follow-up of pregnancy may help early diagnosis and treatment of those complications however there are stills open gap for exact diagnosis.^[1,2]^ The placenta is an endocrine organ that develops during gestation and function to allow transfer of nutrients between mother and fetus. The abnormal placement of placenta in the uterine wall can cause pregnancy related mortalities and morbidities. Moreover, deterioration in blood circulation can histological cause some anomalies in placental structures such as fetal capillaries abnormalities, increased fibrin deposition and fibrotic tissue, degenerated villous structures.^[3,4]^

Preeclampsia (PE) is defined as pregnancy hypertension that affects 3% to 8% of all pregnancies. PE is a leading cause of maternal morbidity and mortality which is characterized after 20^th^ weeaks of gestations. PE is clinically dignosed as systolic hypertension (blood pressure (BP) > 140/90 mm Hg), diastolic hypertension (BP > 90 mm Hg) after 20 weeks of gestation with > 300 mg 24-hour urine protein output. PE is a multifactorial disease that adversely affects kidney and liver function, leading to end-organ injury.^[5]^ In case of severe PE, systolic/diastolic BP pressure ≥ 160/110 mm Hg with low thrombocyte count, increased hepatic aspartate transaminase and alanine transaminase enzymes are observed.^[6]^ Acute necrotizing arteriopathy (acute atherosis) is seen in the spiral arteries in the placentas of women with preeclampsia. These cases are characterized by fibrinoid necrosis in the wall of the vessels, accumulation of lipid-filled cells and mononuclear cell infiltration in the perivascular space. Infarct and retroplacental hematoma mass status in women with preeclampsia is estimated to be a direct result of acute atherosis and related vascular changes.^[7]^

Tumor necrosis factor (TNF)-α is effective on placental invasion and apoptosis.^[8]^ Apoptosis in the placental part increases in pregnancies complicated by preeclampsia or fetal growth retardation.^[9]^ Placental oxidative stress is an effective stimulant for apoptosis. As a result of many studies, the presence of cytokines and receptors of TNF in different human reproductive tissues (amnion and placenta) has been detected.^[10]^

Beclin1 is a 450-amino acid protein that modulate the initiation of autophagy process during starvation. It is also a tumor suppressor gene and takes role in membrane trafficking. Beclin1 interacts with BCL2 to regulate cell survival and death. Expression of Beclin1 is dependent of many factors and involves in neurodegenerative disease and cancers and other disease.^[11,12]^

In this study, we examined the inflammation and autophagy processes between the placentas of preeclamptic and healthy pregnant women immunohistochemically.

## 2. Materials and Methods

In our study, placentas belonging to 20 normotensive and 20 preeclampsia patients were included. Preeclampsia criteria were defined on the basis of American College of Obstetricians and Gynecologists. Placentas were obtained from Dicle University Medical Faculty Hospital Gynecology and Obstetrics Clinic. Ethical approval was obtained from Dicle University, Non-interventional Local Ethical Committee (2023/174). An informed consent form was obtained from all patients participating in the study. Permission was obtained from the local ethics committee for the study. Age (year), gravida, parity, mean systolic pressure (mm Hg), mean diastolic pressure (mm Hg), hemoglobin (g/dL) of pregnant women, platelet (103/µL), glucose, urea, creatinine, alanine aminotransferase enzyme (U/L), aspartate aminotransferase enzyme aspartate aminotransferase (U/L) and 24-hour urine output was recorded and shown in Table 1.

**Table 1:** Patients characteristic and their blood test values were listed.

Parameter	Normotensive (n = 20)	Preeclampsia (n = 20)	Significance
Age	29 (27–36)	30 (26–48)	
Gravida	1 (1–4)	4 (2–9)	
Parity	0 (0–3)	2 (2–5)	
Systolic BP	105 (95–130)	165 (147–187)	*P* < .05
Diastolic BP	70 (60–82)	95 (87–120)	*P* < .05
Hemoglobin	13 (10.1–14.6)	9.7 (7–11.3)	
Platelet	284 (205–372)	276 (195–418)	
Glucose	76 (62–88)	74 (66–96)	
Urea	13 (10–25)	16 (12–32.1)	
Creatinine	0.65 (0.49–0.76)	0.7 (0.54–0.9)	
ALT	16 (10–24)	24 (18–28)	
AST	20 (12–30)	25 (17–30)	
24 h proteinuria	63 (52–75)	752 (340–960)	*P* < .05

Data were shown median (min-max).

ALT = alanine aminotransferase, AST = aspartate aminotransferase, BP = blood pressure.

### 2.1. Histological tissue processing

After the placentas were fixed in 10% formaldehyde, they were passed through the rising alcohol series and included in the routine paraffin-wax tissue protocol. Hematoxylin-Eosin was performed by taking 5 µm sections from paraffin blocks.^[13]^

### 2.2. Immunohistochemical examination

The obtained placental sections were washed with distilled water to perform immunohistochemistry. Then, for the antigen application process, the application was carried out by waiting at 700 W in a microwave oven for 10 minutes in citrate buffer solution with pH: 6.0. After this process, the obtained sections were allowed to cool for up to 25 minutes at room temperature and washed in distilled water for 2 × 4 minutes. As a result of these procedures, 3% hydrogen peroxide was used for 10 minutes in order to apply endogenous peroxidase blockade. The tissues we obtained were both washed with distilled water and washed with PBS. After performing the washing procedures, the sections obtained from the sections were incubated overnight at + 4°C with mouse monoclonal anti-TNF-α and anti-Beclin1 antibodies (AFG Bioscientific, 1:100). The sections that were kept the next day were cleaned with PBS and applied with a secondary antibody solution (Biotinylated Goat Anti-Mouse, Lab Vision) for 20 minutes. After PBS treatment, streptavidin peroxidase solution (Streptavidin Peroxidase, Lab Vision) was applied for 15 minutes. After the obtained sections were washed 3 times in PBS, DAB chromogen solution was applied for 8 minutes. Afterwards, the sections were washed with distilled water and counterstained with Harris hematoxylin for 2 minutes. The obtained sections were then allowed to be viewed under a light microscope with the imager A2 Zeiss.^[14,15]^

### 2.3. Statistical analysis

In order to perform the statistical analysis of the obtained data, IBM SPSS Statistics version 25 software was used and evaluated. The obtained data were analyzed by applying normality tests. In order to make a better evaluation of the obtained data, the Mann–Whitney *U* test was used to make a pairwise comparison in the SPSS program. A *P* value <.05 was accepted as statistically significant at the threshold of evaluation and results obtained.

## 3. Results

**Figure 1.**
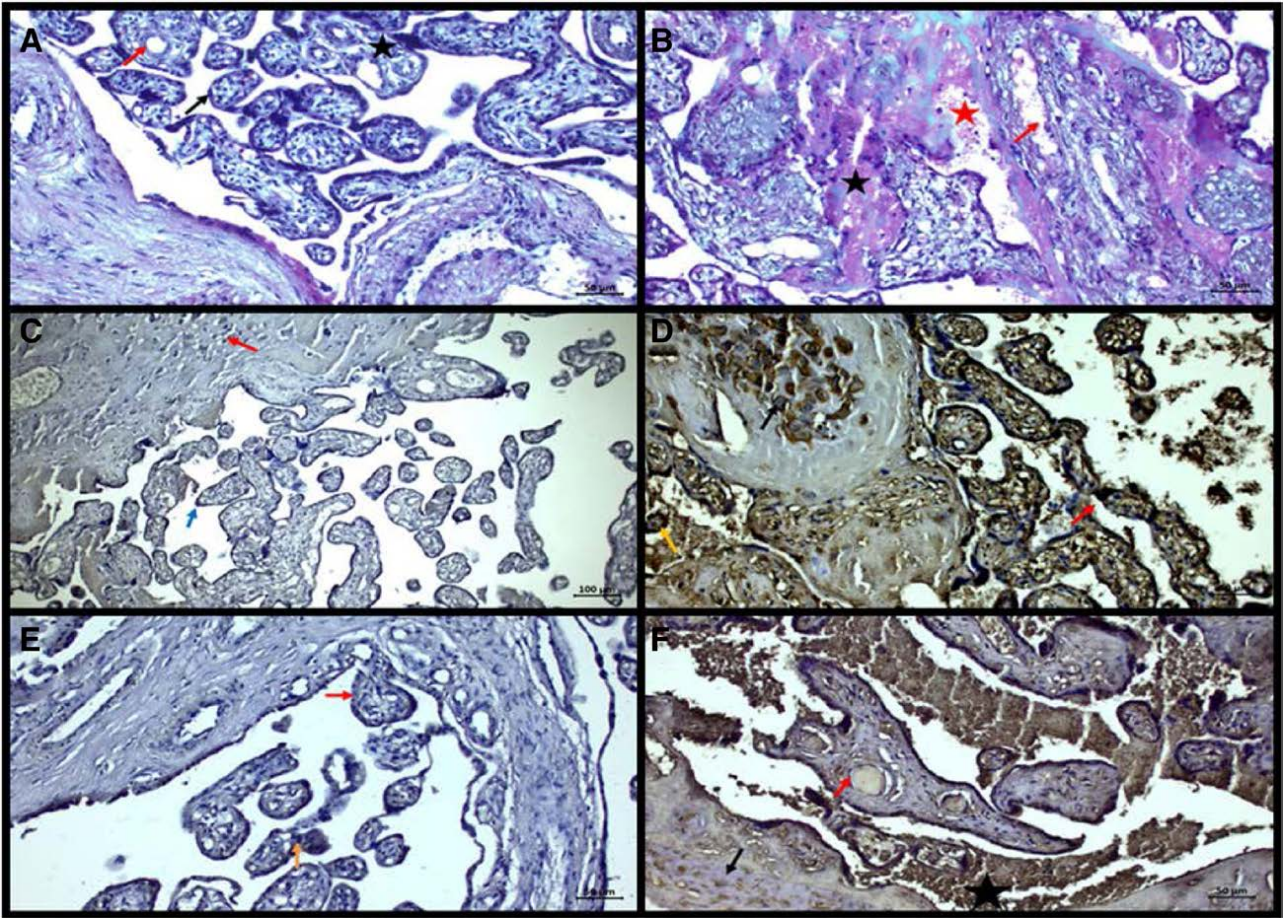
(A) Normal villi structure (black arrow), vascular structures (red arrow) and connective tissue (star), (B) Pyknosis (black arrow) in decidual cells, increase in fibrinoid tissue (star), syncytial, vascular dilatation (red arrow) and cell infiltration (red star), (C) negative Beclin1 expression in decidual cells (red arrow) in the maternal region, positive Beclin1 expression in terminal nodes (blue arrow), (D) Positive Beclin1 reaction around the decidual membrane (black arrow), syncytial nodes and bridges (red arrow), endothelial cells and intervillous spaces (yellow arrow), (E) Negative TNF-α in root villi, floating villi (red arrow), syncytial bridges (orange arrow) in the maternal region, and (F) Positive TNF-α expression in decidua cells (black arrow), vessel wall endothelium (red arrow), and leukocyte structures (asterisk). TNF = tumor necrosis factor.

Figure 1 shows histochemical and immunohistochemical staining. Control Hematoxylin-Eosin staining: Root and floating villi were regular, syncytial node was less, vascular structures and connective tissue were histologically normal. No hemorrhage was observed in the intervillous area (Fig. 1A). Preeclampsia Hematoxylin-Eosin staining: Pyknosis in decidual cells, increase in fibrinoid tissue, increase in syncytial nodes and bridges, hyperplasia in vascular endothelium, vascular dilatation and increase in cell infiltration in connective tissue cells were observed (Fig. 1B).

### 3.1. Control Beclin1 immunostaining

Except for some syncytial cells in roots and floating villi, especially in areas where the maternal region is located, the Beclin1 reaction was generally evaluated as negative. Some syncytial areas and bridges were also evaluated as negative for Beclin1 reaction (Fig. 1C). Preeclampsia Beclin1 immunostaining: It was observed that the Beclin1 reaction was evident especially around the decidual membrane. It was observed that the Beclin1 reaction was increased in the syncytial nodes and bridges, and the Beclin1 reaction was positive in the chorionic villi, especially in the root villi and the connective tissue cells in the inner parts of the floating villi, and in some Hoffbauer cells. Again, positive Beclin1 reaction was observed in endothelial cells and intervillous areas (Fig. 1D).

### 3.2. Control TNF-α immunostaining

In the section passing through the maternal region, clearly negative TNF-α expression was observed in decidual cells. It is negative in syncytial regions as well as in some cytotrophoblast cells. However, TNF-α expression was positive in some end nodes, especially towards the intervillous area. In general terms, TNF-α reaction was found to be negative (Fig. 1E).

### 3.3. Preeclampsia TNF-α immunostaining

TNF-α reaction was observed in parallel with significant degenerative changes in decidua cells. TNF-α reaction was positive in the vessel wall, especially in endothelial cells, in leukocyte structures in intervillous areas and in connective tissue cells inside the villi. It was observed that TNF-α reaction was positive with degenerative changes in the regions (Fig. 1F).

## 4. Discussion

The most important difference between preeclampsia and normal pregnancy is that cytotrophoblasts invade spiral arteries both in decidual sections and myometrium and increase uteroplacental blood supply by decreasing vascular resistance in normal pregnancies, whereas in preeclampsia cytotrophoblasts invade spiral arteries only in decidual section and cannot penetrate the myometrium, there are differences in hypoperfusion and remodeling of vessels In preeclampsia, it has been reported that spiral artery invasion is incomplete in many regions of the placenta and endovascular cytotrophoblasts are very few.^[16,17]^

Preeclampsia can induce many cellular events such as inflammatory pathway and oxidative stress mechanism. Some authors have found a reduced degree of trophoblast invasion in both the spiral arteries and the myometrium in severe preeclampsia.^[18]^ Akhlaq et al^[19]^ studied placentas of patients with preeclampsia. They found that histopathological alterations can vary depending on the severity of preeclampsia. They observed hypertrophic muscle and decidual cells, in the maternal-fetal region, hyperplasic placental villi, elevated number of syncytial nodes and leukocyte infiltration in connective tissue, and accumulation of chorionic villi in syncytial regions of chorionic villi. In our study, in control group, root and floating villi were normal in histological perspectives, syncytial node number was low, vessels were normal with connective tissue. No hemorrhage was observed in the intervillous area (Fig. 1A). In preeclampsia group, decidual cell degeneration and fibrinoid accumulation increased. Vascular dilatation and congestion with mononuclear cell infiltration were observed (Fig. 1B).

Various studies have shown that apoptosis is increased in pregnancies complicated by some pathologies such as preeclampsia, fetal growth restriction and diabetes.^[20]^ Unlike apoptotic tissue, necrotic tissues contribute to the pathophysiology of preeclampsia by forming an inflammatory response when taken up by endothelial cells.^[21]^ These conditions fail to explain the role of apoptosis in the development of placental pathology.^[22]^ In our study, Beclin1 reaction was generally negative in control group (Fig. 1C). In preeclampsia group, Beclin1 reaction was increased in decidual cells, syncytial nodes and bridges and in chorionic villi and in some Hoffbauer cells (Fig. 1D).

Placental trophoblastic cells and fetoplacental macrophages normally express TNF-α, which leads to endothelial cell activation and dysfunction. Basu et al,^[23]^ in their study, stated that the failure of the increase in TNF-α protein expression in trophoblast cells in the second trimester of normal pregnancy alters or disrupts the remodeling process of the spiral arteries, prevents the placental angiogenic-antiangiogenic balance, and may induce syncytiotrophoblast stress. It has been suggested that migration activity, increase in syncytial node and endocrine functions after high TNF-α levels in risky pregnancies such as gestational diabetes, mild preeclampsia, and severe preeclampsia affect trophoblast biology.^[24]^ In our study, in control group, TNF-α expression was mainly negative but only in some decidual cells. (Fig. 1E). In preeclampsia, TNF-α reaction was observed in degenerated decidua cells, in leukocytes and in villi (Fig. 1F).

## 5. Conclusion

In conclusion, in preeclampsia placentas, degenerative changes in decidua cells in the maternal region continued in placental villi and an increase in inflammation was observed in the intervillous area. It was observed that the apoptotic change due to inflammation increased and the angiogenic effect changed. It was thought that Beclin1 TNF-α signals could be used as a marker in affecting the fetal structure of blood flow in preeclamptic placentas.

## Author contribution

**Conceptualization:** Firat Aşir, Veysel Toprak, Senem Alkan Akalin, Engin Deveci.

**Data curation:** Ece Öcal.

**Formal analysis:** Ece Öcal, Veysel Toprak.

**Funding acquisition:** Veysel Toprak, Engin Deveci.

**Investigation:** Senem Alkan Akalin, Engin Deveci.

**Methodology:** Ece Öcal.

**Project administration:** Firat Aşir, Senem Alkan Akalin.

**Resources:** Veysel Toprak, Engin Deveci.

**Software:** Ece Öcal.

**Supervision:** Firat Aşir.

**Validation:** Ece Öcal, Engin Deveci.

**Visualization:** Senem Alkan Akalin.

**Writing – original draft:** Firat Aşir, Veysel Toprak, Senem Alkan Akalin.

**Writing – review & editing:** Firat Aşir, Senem Alkan Akalin.
